# P21 Dalbavancin as long-term suppressive therapy for complex deep-seated infections: a retrospective service evaluation

**DOI:** 10.1093/jacamr/dlag102.027

**Published:** 2026-06-26

**Authors:** Jaffrey Thavasingh, Fiona Elliott, Darshini Reyes, Ivan Tonna, Vhairi Bateman, Helen Callaby

**Affiliations:** Aberdeen Royal Infirmary, Aberdeen, UK; Aberdeen Royal Infirmary, Aberdeen, UK; Aberdeen Royal Infirmary, Aberdeen, UK; Aberdeen Royal Infirmary, Aberdeen, UK; Aberdeen Royal Infirmary, Aberdeen, UK; Aberdeen Royal Infirmary, Aberdeen, UK; University of Aberdeen, Aberdeen, UK

## Abstract

**Background:**

Dalbavancin is a novel long-acting lipoglycopeptide antibiotic approved for acute bacterial skin and skin structure infections. Its unique pharmacokinetic profile has led to off-label use as suppressive therapy for deep-seated infections in some centres. This service evaluation reviews the use of suppressive Dalbavancin in a tertiary infection centre in Scotland.

**Objectives:**

This service evaluation aimed to characterize the patient population receiving dalbavancin suppression and evaluate treatment outcomes as well as highlight areas for improvement in clinical practice.

**Methods:**

NHSG Caldicott approval was granted for this study. No funding was required. This was a retrospective case notes review of all patients receiving suppressive dalbavancin (defined as >6 months duration with no curative intent) through the NHS Grampian OPAT service from January 2022 to November 2024. Data was analysed with descriptive statistics using excel.

**Results:**

17 patients received suppressive therapy; 9 males and 8 females with a median age of 63 years (range 18-74). Treatment duration varied from 6 months to over 6 years. Indications included chronic prosthetic joint infections (9/17, 53%), chronic osteomyelitis with no surgical available options (6/17, 35%), infected vascular prosthetic grafts with no surgical available options (1/17, 6%), and recurrent cellulitis in patients with underlying chronic conditions predisposing to an increased risk of reinfections (1/17, 6%). Notably, (6/17, 35%) had failed previous suppressive therapies due to intolerance (3) or relapse (3), while (7/17, 41%) lacked documented rationale for choosing dalbavancin. (3/17 18%) remained relapse-free; (2/17, 12%) achieved suppression until definitive surgery; (4/17, 24%) experienced breakthrough infections (one requiring ICU care for septic shock, however, with gram negative infection); and (3/17, 18%) died (one directly OM infection-related).

**Conclusions:**

This evaluation demonstrates both the potential and challenges of dalbavancin as suppressive therapy. While some patients achieved good outcomes, the small sample size and multimorbid population with complex infection preclude definitive efficacy conclusions. Key limitations include the retrospective design, inconsistent monitoring, and lack of standardized outcome measures.

